# Prevalence and phylogenetic analysis of porcine circovirus type 2 (PCV2) and type 3 (PCV3) in the Southwest of China during 2020–2022

**DOI:** 10.3389/fvets.2022.1042792

**Published:** 2022-11-24

**Authors:** Yanting Yang, Tong Xu, Jianhua Wen, Luyu Yang, Siyuan Lai, Xiangang Sun, Zhiwen Xu, Ling Zhu

**Affiliations:** ^1^College of Veterinary Medicine, Sichuan Agricultural University, Chengdu, China; ^2^College of Veterinary Medicine Sichuan Key Laboratory of Animal Epidemic Disease and Human Health, Sichuan Agricultural University, Chengdu, China

**Keywords:** prevalence, genotype, phylogenetic analysis, PCV2, PCV3

## Abstract

**Introduction:**

Porcine circovirus type 2 (PCV2) is considered one of the viruses with substantial economic impact on swine industry in the word. Recently, porcine circovirus type 3 (PCV3) has been found to be associated with porcine dermatitis and nephropathy syndrome (PDNS)-like disease. And the two viruses were prone to co-infect clinically.

**Methods:**

To further investigate the prevalence and genetic diversity of the two viruses, 257 pig samples from 23 different pig farms in southwest China with suspected PCVAD at different growth stages were analyzed by real-time PCR between 2020 and 2022 to determine the presence of PCV2 and PCV3.

**Results:**

Results showed high prevalence of PCV2 and PCV3: 26.46% samples were PCV2 positive and 33.46% samples were PCV3 positive. The coinfection rate was doubled from 2020 (5.75%) to 2022 (10.45%). Subsequently, the whole genome sequences of 13 PCV2 and 18 PCV3 strains were obtained in this study. Of these, 1 strain was PCV2a, 5 strains were PCV2b and 7 strains were PCV2d, indicating that PCV2d was the predominant PCV2 genotype prevalent in the Southwest of China.

**Discussion:**

In addition, the phylogenetic analysis of PCV3 showed high nucleotide homology (>98%) between the sequences obtained in this study and reference sequences. And 3 mutations (A24V, R27K and E128D) were found in PCV3 antibody recognition domains, which might be related to the mechanism of viral immune escape. Thus, this study will enhance our understanding of the molecular epidemiology and evolution of PCV2 and PCV3, which are conducive to the further study of the genotyping, immunogenicity and immune evasion of PCVs.

## Introduction

Porcine circoviruses (PCVs) are circular single-stranded DNA virus, belonging to the genus Circovirus of the family Circoviridae, which causes disease in birds, dogs, penguins, foxes, bears and pigs (1). Four types of PCV have been identified, namely PCV1, PCV2, PCV3 and PCV4 (2–5). PCV1 was first detected in 1974 as a cell culture contaminant and was not pathogenic to pigs (6). In 1998, Allan identified a circovirus structurally similar to PCV1, but with <80% homology, and named it PCV2 (3). Unlike PCV1, PCV2 has long been recognized as the major pathogen causing PCVD/PCVAD (Porcine Circovirus Associated Disease), characterized by clinical or subclinical infection with PCV2 in pigs (7). The symptoms include porcine dermatitis and nephropathy syndrome (PDNS), postweaning multisystemic wasting syndrome (PMWS) and porcine respiratory disease complex (PRDC) (8). In 2015, PCV3 was first detected in the U.S and could cause reproductive disorders, skin diseases, and multisystem inflammation in pigs (4, 9). In the last 2 years, a new circovirus was detected, and the affected pigs showed respiratory disorders, intestinal symptoms, and porcine dermatitis nephrotic syndrome (PDNS), named PCV4 (10).

PCV2 is a globally transmitted frequent pathogen and its genome is gradually undergoing genetic mutations. Its viral genome contains 1,766–1,768 nucleotides, with 11 overlapping and mosaic open reading frames. Among them, ORF1 and ORF2 are the two most dominant open reading frames, ORF1 encodes two replication-related proteins, Rep and Rep' (11), ORF2 encodes the viral structural protein (Cap), and the Cap protein is also the only immunogenic protein of PCV2 virus (12). ORF2 is highly variable and commonly used in phylogenetic analyses of PCV2. At present, PCV2 was classified into eight genetic isoforms (PCV2a - PCV2h) (13), and coinfection might occur between the different subtypes, leading to recombination of the ORF1 and ORF2 genes, resulting in new genotypes. Since the discovery of PCV2, there have been two shifts in its prevalent genotype: from PCV2a to PCV2b around 2000; and from PCV2b to PCV2d around 2012 (12).

Since its discovery, PCV3 has been prevalent in several countries including China, Japan, Korea, Sweden, Russia, Thailand, Malaysia and Italy (14, 15). The PCV3 genome contains 1999–2001 nucleotides and is similar to PCV2 in that it also has two major open reading frames, ORF1 and ORF2, encoding the Rep and Cap proteins, respectively, which share only about 40% homology with PCV2 (14). The Cap protein of PCV3 has two amino acid sites that are prone to mutation (A24V and R27K), and PCV3 can be classified into three subtypes PCV3a-3c based on these two mutations (16). The rate of genetic evolution of PCV3 was found to be much lower than that of PCV2, about 10-5 substitutions/locus/year (17).

PCV2 and PCV3 are pathogenic to the swine industry which has brought huge economic losses. Co-infections of viruses play a critical role in pathogenicity and clinical symptoms (18). Therefore, monitoring the prevalence and genetic characteristics of both viruses is necessary. Prior to this, there have been partially reports on the prevalence and genetic variation of PCV2 and PCV3 in China (16–21), but the information in the Southwest of China was limited. In this trial, real-time PCR was used to detect the prevalence of PCV2 and PCV3 in the Southwest of China from 2020 to 2022. Based on molecular epidemiology and phylogenetic analysis, this study will deepen our research on the genetic characteristics of PCV2 and PCV3 viruses.

## Materials and methods

### Sample collection

**Figure 1:**
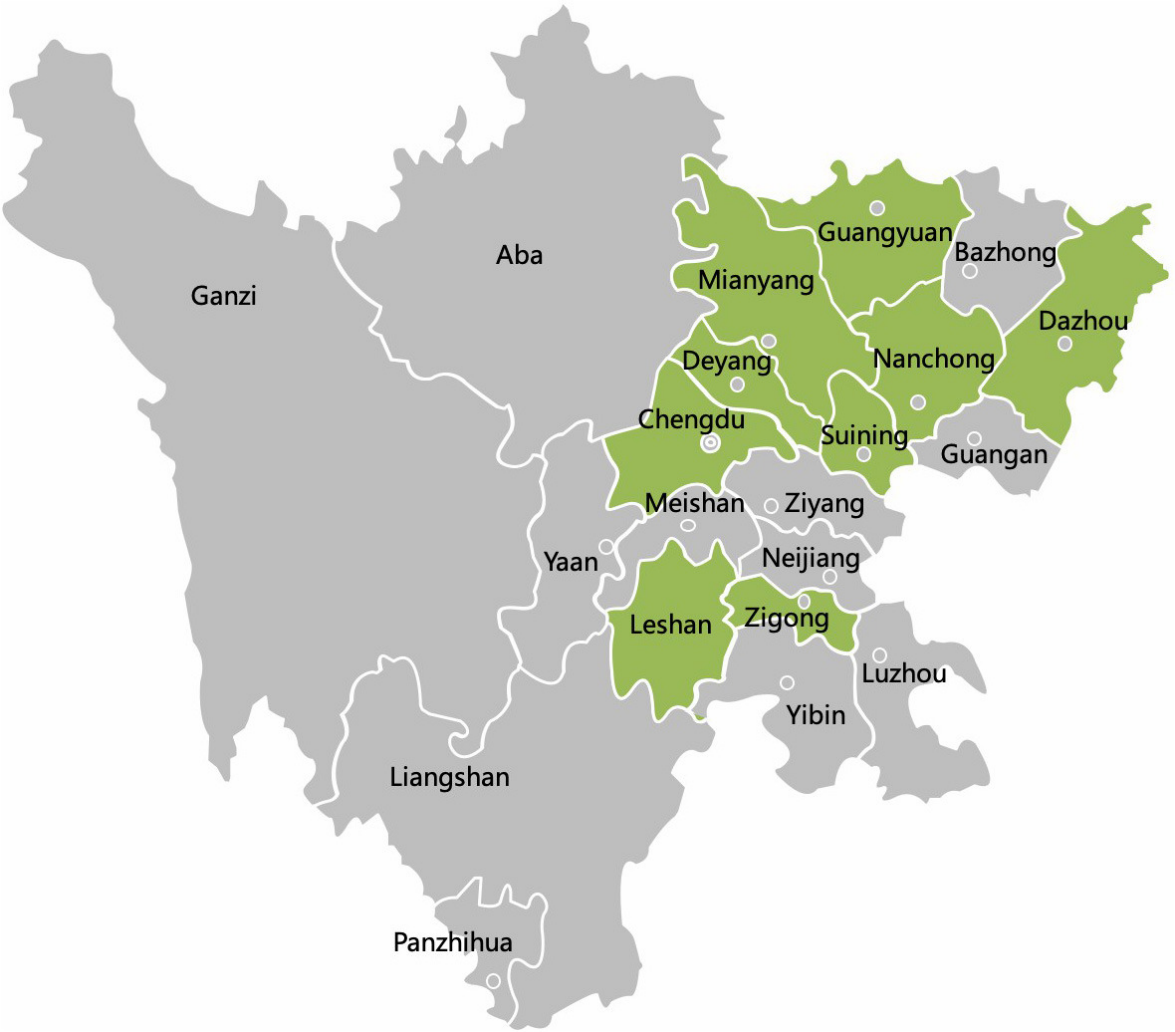
The geographical distribution of 257 samples in Sichuan Province, China. Qionglai, Dayi, Pengzhou, and Jianyang are all parts of Chengdu. The cities with samples collected are marked with green color.

A total of 257 diagnostic samples from our laboratory were randomly collected from 23 different pig farms in 12 cities in Sichuan province during September 2020 and May 2022 (Figure 1). Of these, 14/23 were vaccinated against PCV2. Different sample types including heart, lung, liver, spleen, kidney, lymph nodes, intestine, nasal swabs and serum were collected. The animals were of different ages. Among diagnostic samples, 72.37% (186/257) were suspected to have respiratory syndrome pathogen, and 27.63% (71/257) were for pathogens of enteric diarrhea. Samples were processed following standard operating procedures.

### Detection of PCV2 and PCV3

Total viral DNA was extracted from the collected samples using the FastPure Cell/Tissue DNA Isolation Mini Kit (Cofitt, Hangzhou, China). The DNA samples were detected for PCV2 and PCV3 positivity by a validated TB Green II-based multiplex real-time PCR assay as described previously (22). The results were analyzed with Bio-Rad CFX Manager 4.1.

### Detection of other pathogens

In addition to PCV2 and PCV3 detection, according to the different symptoms of the clinical cases from which the samples were derived, porcine epidemic diarrhea virus (PEDV), porcine deltacoronavirus (PDCoV), porcine rotavirus (PoRV), porcine transmissible gastroenteritis virus (TGEV), porcine reproductive and respiratory syndrome virus (PRRSV), swine influenza virus (SIV) and (MHP) were also detected. The assays used were as described previously (23–26).

### Whole genome sequencing of PCV2 and PCV3

To obtain full-length sequences of PCV2 and PCV3 for phylogenetic analysis, PCR primers were utilized for whole genome sequencing as previously described (15, 27). Briefly, the 20-μL PCR reaction mixture contained 10 μL of 2 × PrimeSTAR Max Premix (Takara), 2 μL of sample DNA, 0.6 μL of each primer (25 μM), and 6.8 μL of ddH2O. The PCR reaction of PCV2 was executed by 35 cycles of 98°C for 10s, 57°C for 5s, and 72°C for 30s. Amplification conditions of PCV3 were similar to PCV2, excepting that the annealing time was 15s for the PCV3 primer sets.

The purified PCR products cloned into pMD18-T Cloning Vector (Takara, Dalian, China), and propagated in DH5α competent cells (Takara) following the manufacturer's instructions. Each PCR product was cloned three times and independently sequenced by Sangon Biotech Shanghai Co, Ltd. All sequencing reactions were performed in duplicate. The whole genome sequences of PCV2 were obtained in one shot by sequencing. The genomic sequences of PCV3 were assembled from the sequenced overlapping sequences by the EditSeq program of the LaserGene software package (DNASTAR, Inc., Madison, WI).

### Sequence analyses

The reference strains and genomic sequences of PCV2 and PCV3 strains obtained in this study were shown in Supplementary Table S1. The complete coding sequence (ORF1 + ORF2) of PCV3 was analyzed. The coding sequence was divided into two sequences: ORF1 (Rep) and ORF2 (Cap), after the non-coding region was deleted. Since ORF2 was in the opposite direction, we reversed it and then spliced ORF1 and ORF2. The PCV3 subtypes were proposed by Li (28). The complete coding sequences of PCV3 and the ORF2 of PCV2 strains were compared using the Clustal W function of the Molecular Evolutionary Genetics Analysis (MEGA) software (version 7.0). Phylogenetic trees were generated by the maximum likelihood (ML) method in MEGA 7.0 with a p-distance model, and a bootstrap of 1000 replicates.

### Ethics statement

All samples used in the study were pig samples, did not involve any human samples. Written informed consent was obtained from animal owners for this study. All experimental procedures were reviewed and approved by the Sichuan provincial laboratory management committee [License No: SYXK (chuan) 2019–187].

## Results

### Prevalence of PCV2 and PCV3 during 2020–2022

#### Overall prevalence of PCV2 and PCV3

Of the 257 clinical samples detected, the positive rates of PCV2 and PCV3 were 26.46% (68/257) and 33.46% (86/257), respectively, and the coinfection rate of PCV2 and PCV3 was 8.95% (23/257). When the data was analyzed by year, prevalence of PCV3 in samples was 33.33% (26/87) in 2020, 35.92% (35/103) in 2021 and 37.31% (25/67) in 2022, where the prevalence rates were higher for each corresponding year compared to PCV2: 24.14% (21/87) in 2020, 26.13% (27/103) in 2021 and 29.85% (20/67) in 2022. The prevalence of PCV2 and PCV3 has gradually increased in the past 3 years, and the coinfection rate of PCV2 and PCV3 also increased rapidly, from 5.75% (5/87) in 2020, to 9.71% (10/103) in 2021 then to 10.45% (7/67) in 2022 (Table 1).

**Table 1:** The prevalence rates of PCV3 and PCV2 from swine specimens collected in the Southwest of China.

**Year**	**PCV2 positive/total (%)**	**PCV3 positive/total (%)**	**Coinfection positive/total (%)**
2020	21/87(24.14%)	26/87(33.33%)	5/87(5.75%)
2021	27/103(26.13%)	35/103(35.92%)	10/103(9.71%)
2022	20/67(29.85%)	25/67(37.31%)	7/67(10.45%)
**Total**	68/257(26.46%)	86/257(33.46%)	23/257(8.95%)

#### Geographic distribution of PCV2 and PCV3

The clinical samples in the trial were collected from 12 cities during 2020–2022, which were summarized in Table 2. Both PCV2 and PCV3 were identified from samples of 12 cities. It demonstrated that PCV2 and PCV3 were prevalent in the Southwest of China from 2020 to 2022, and the positive rate of PCV3 was higher than that of PCV2 in any region. Among the 23 pig farms in these 12 cities, 69.57% (16/23) were positive for PCV2 and 47.83% (11/23) were positive for PCV3 and 39.13% (9/23) were diagnosed as coinfection of PCV2 and PCV3.

**Table 2:** The prevalence rates of PCV2 and PCV3 in different cities in the Southwest of China during 2020–2022.

**Farms**	**Cites**	**PCV2 positive case rate (%)**			**PCV3 positive case rate (%)**		
		**2020**	**2021**	**2022**	**2020**	**2021**	**2022**
Farm 1	Mianyang	1/3 (33.33%)	2/6 (33.33%)	1/4 (25%)	2/3 (66.67%)	2/6 (33.33%)	2/4 (50%)
Farm 2	Mianyang	1/5 (20%)	1/7 (14.29%)	0/3 (0%)	1/5 (20%)	2/7 (28.57%)	1/3 (33.33%)
Farm 3	Mianyang	2/8 (25%)	3/9 (33.33%)	2/7 (28.57%)	3/8 (37.5)	5/9 (55.56%)	3/7 (42.86%)
Farm 4	Mianyang	2/7 (28.57%)	2/6 (33.33%)	1/3 (33.33%)	3/7 (42.86%)	2/6 (33.33%)	1/3 (33.33%)
Farm 5	Suining	1/7 (14.29%)	1/5 (20%)	0/2 (0%)	2/7 (28.57%)	1/5 (20%)	1/2 (50%)
Farm 6	Suining	1/5 (20%)	1/7 (14.29%)	0/2 (0%)	1/5 (20%)	2/7 (28.57%)	0/2 (0%)
Farm 7	Suining	2/9 (22.22%)	3/11 (27.27%)	1/3 (33.33%)	1/9 (11.11%)	3/11 (27.27%)	2/3 (66.67%)
Farm 8	Suining	1/6 (16.67%)	2/6 (33.33%)	1/2 (50%)	2/6 (33.33%)	2/6 (33.33%)	1/2 (50%)
Farm 9	Jianyang	1/3 (33.33%)	0/2 (0%)	0/1 (0%)	0/3 (0%)	1/1 (100%)	0/1 (0%)
Farm 10	Jianyang	0/1 (0%)	-	0/1 (0%)	0/1 (0%)	0/1 (0%)	1/1 (100%)
Farm 11	Deyang	0/1 (0%)	1/3 (33.33%)	-	1/1 (100%)	0/3 (0%)	-
Farm 12	Qionglai	2/4 (50%)	1/4 (25%)	1/3 (33.33%)	1/4 (25%)	2/4 (50%)	2/3 (66.67%)
Farm 13	Qionglai	1/3 (33.33%)	1/4 (25%)	1/4 (25%)	2/3 (66.67%)	1/4 (25%)	0/4 (0%)
Famr 14	Qionglai	0/2 (0%)	1/3 (33.33%)	1/3 (33.33%)	1/2 (50%)	1/3 (33.33%)	2/3 (66.67%)
Farm 15	Dayi	-	1/4 (25%)	-	-	1/4 (25%)	-
Farm 16	Pengzhou	1/3 (33.33%)	1/1 (100%)	0/2 (0%)	0/3 (0%)	0/1 (0%)	0/2 (0%)
Farm 17	Pengzhou	1/4 (25%)	0/2 (0%)	1/4 (25%)	2/7 (28.57%)	0/2 (0%)	0/4 (0%)
Farm 18	Dazhou	0/1 (0%)	1/3 (33.33%)	2/5 (40%)	0/1 (0%)	1/3 (33.33%)	1/5 (20%)
Farm 19	Guangyuan	0/1 (0%)	1/5 (20%)	2/6 (33.33%)	1/1 (100%)	2/5 (40%)	3/6 (50%)
Farm 20	Nanchong	1/3 (33.33%)	1/5 (20%)	3/5 (60%)	2/3 (66.67%)	2/5 (40%)	2/5 (40%)
Farm 21	Zigong	1/3 (33.33%)	1/3 (33.33%)	-	0/3 (0%)	2/3 (66.67%)	-
Farm 22	Zigong	1/5 (20%)	1/3 (33.33%)	0/2 (0%)	1/5 (20%)	1/3 (33.33%)	1/2 (50%)
Farm 23	Leshan	1/3 (33.33%)	1/4 (25%)	3/5 (60%)	0/3 (0%)	2/4 (50%)	2/5 (40%)
	**Total**	21/87 (24.14%)	27/103 (26.13%)	20/67 (29.85%)	26/87 (33.33%)	35/103 (35.92%)	25/67 (37.31%)

–, No sample available.

#### Prevalence of PCV2 and PCV3 in different sample types

In this study, a total of 9 different types of sample were testing for PCV2 and PCV3, and the positive rates were shown in Table 3. Among them, 42.12% (109/257) were porcine serum, of which 26.61% (29/109) were PCV2 positive and 33.94% (37/109) were PCV3 positive. In the tissue samples, the positive rates of PCV2 and PCV3 were 40% (8/20) and 45% (9/20) in lymph node, 37.84% (14/37) and 48.65% (17/37) in spleen, respectively, which were higher than those in other tissues. Besides, Table 3 illustrated that PCV3 was mostly detected in spleens and PCV2 was more common in lymph node. Meanwhile, the coinfection rates in spleen and lymph node were also higher than those in other sample types.

**Table 3:** Positive rates of PCV3 and PCV2 in different types of swine samples during 2020–2022.

**Sample type**	**PCV2 positive rate positive/total (%)**	**PCV3 positive rate positive/total (%)**	**Coinfection rate positive/total (%)**
Heart	0/5 (0%)	1/5 (20%)	0/5 (0%)
Lung	8/33 (24.24%)	10/33 (30.30%)	3/33 (9.09%)
Liver	1/7 (14.29%)	0/7 (0%)	0/7 (0%)
Spleen	14/37 (37.84%)	17/37 (48.65%)	4/37 (10.81%)
Kidney	5/29 (17.24%)	7/29 (24.14%)	2/29 (6.90%)
Lymph node	8/20 (40%)	9/20 (45%)	2/20 (10%)
Intestine	1/6 (16.67%)	1/6 (16.67%)	0/6 (0%)
Nose swabs	2/11 (18.18%)	4/11 (27.27%)	1/11 (9.09%)
Serum	29/109(26.61%)	37/109 (33.94%)	11/109 (10.09%)
**Total**	68/257 (26.46%)	86/257 (33.46%)	23/257 (8.95%)

#### Coinfection with other porcine viruses

In addition to the coinfection of PCV2 and PCV3 mentioned above, we also investigated the coinfection rates of PCVs with other common pathogens in pigs. Of the 56 cases of enterovirus infection including PEDV, PDCoV, PoRV and TGEV, 16. 07% (9/56) were PCV2 positive and 21.43% (12/56) were PCV3 positive. In the 89 respiratory virus infection cases including PRRSV, SIV and MHP, 24.72% (22/89) were PCV2 positive and 34.83% (31/89) were PCV3 positive. These results showed that the coinfection rate with other pathogens of PCV3 is higher than that of PCV2, and the coinfection of PCVs with respiratory pathogens is higher than that of intestinal pathogens, which might be related to the pathogenic mechanism of PCVs.

### Sequence analysis of PCV2 and PCV3 prevalent in southwest Chine during 2020–2022

#### Sequences to submit

From this study, a total of 17 PCV2 and 23 PCV3 sequences were obtained. Among them, the unique 13 PCV2 and 18 PCV3 whole genome sequences were generated and deposited in the NCBI GenBank with accession number as follows: OP055737-OP055748(PCV2), OP189294(PCV2), ON989005(PCV3), ON586851(PCV3), and OP066314-OP066329(PCV3).

#### Genetic characteristics of PCV2

**Figure 2:**
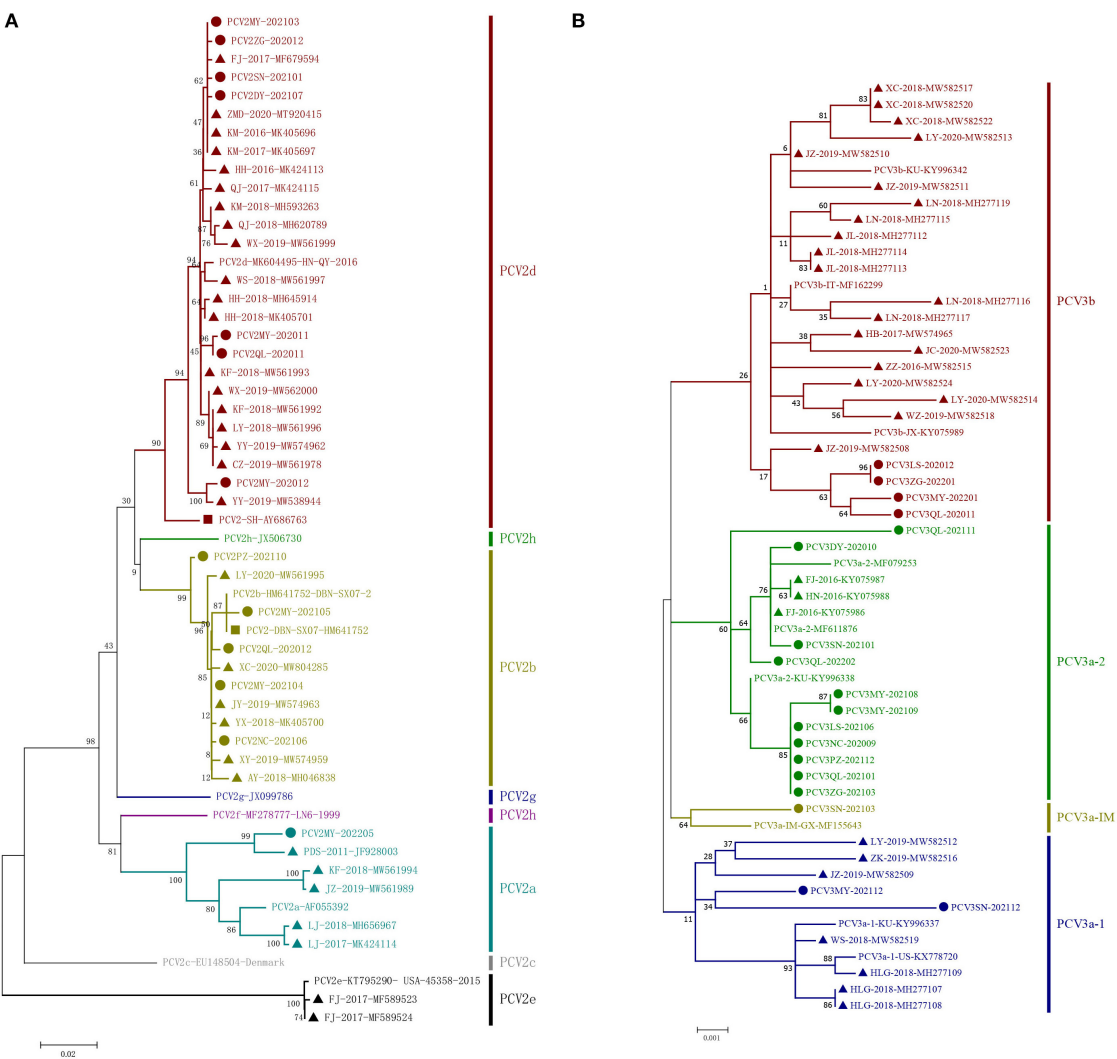
Phylogenetic trees based on the ORF2 gene of PCV2 and the complete coding sequences (ORF1 + ORF2) of PCV3. The ML tree was constructed with a p-distance model and bootstrapping at 1,000 replicates. **(A)**: PCV2. **(B)**: PCV3. The PCV3 subtypes were proposed by Li et al. (28). The strains sequenced in this study are marked with circles; the Chinese reference sequences are marked with triangle; the vaccine strains are marked with square. The different genotypes are represented by different colors as indicated in the figures.

A phylogenetic tree was constructed based on ORF2 of 13 unique strains and 24 genotypic reference strains (PCV2a-h) (Figure 2A). The results showed that 1 strain of PCV2 belonged to PCV2a, 5 strains belonged to PCV2b and 7 strains belonged to PCV2d.

**Figure 3:**
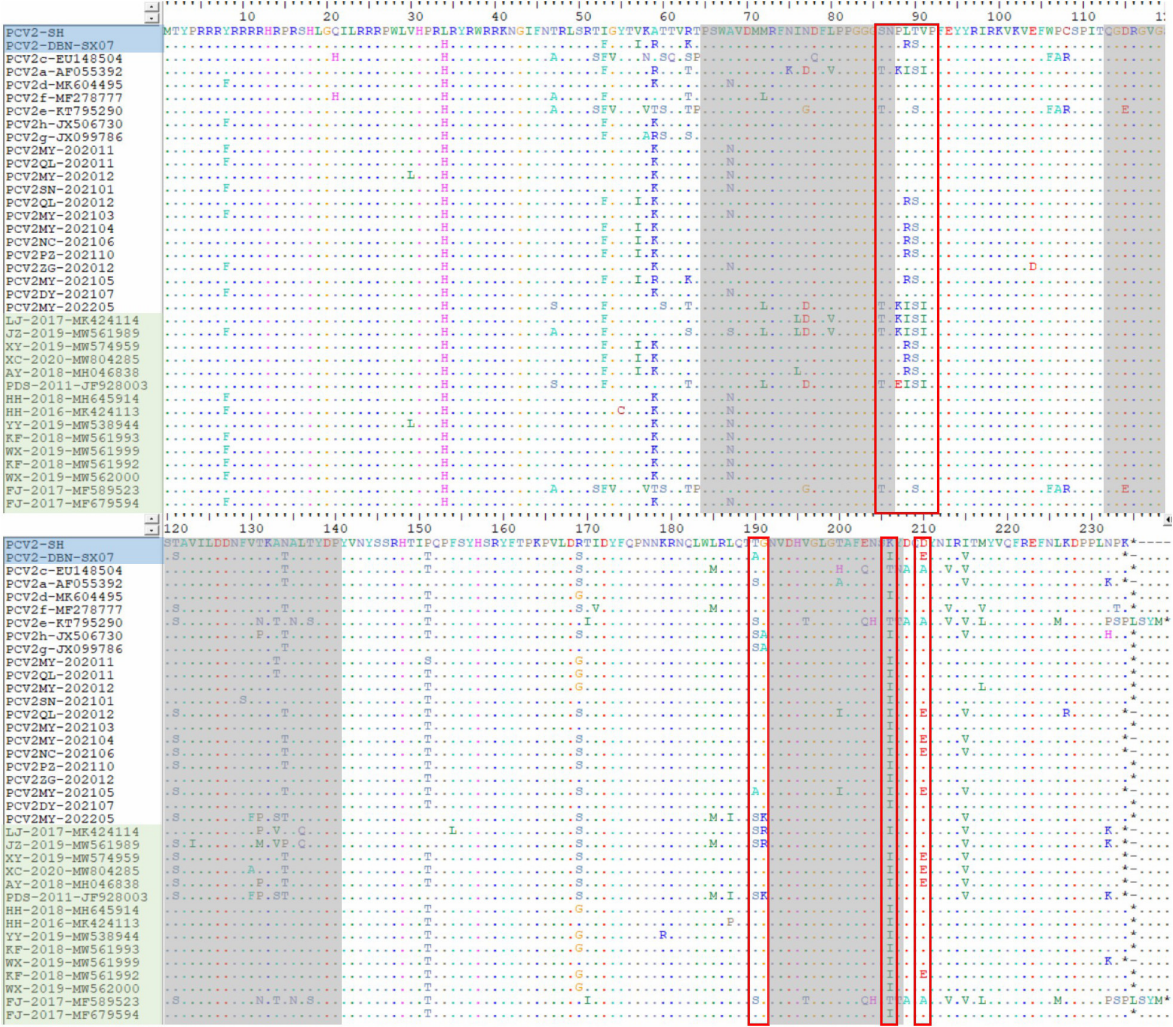
The unique amino acid sequences encoded by ORF2 of the PCV2 strains. The red open boxes show the typical motifs of ORF2 gene. The gray areas represent the epitope region reported in previous studies, including A (65–87), B (113–139), C (193–207) and D (227–233). The blue area represents the vaccine strains and the green area represents the Chinese reference sequences.

Sequence analysis indicated that the 13 strains shared 94.7–99.9% identity of their whole genomes, 89.9–99.9% identity of ORF2 and 97.1–100% of ORF2. Compared with 24 reference strains deposited in GenBank database, the 13 strains shared 90.9–99.9% identity of their whole genomes, 80.7–99.9% identity of ORF2 and 96.5–100% identity of ORF1. Among the 13 unique PCV2 sequences, 11 unique capsid (Cap) proteins and 6 unique replicase (Rep) proteins were generated based on nucleotide mutations. At the amino acid level, the similarities between the 11 Cap proteins were 88.1–99.6%, while the similarities between them and the 24 PCV2 reference genomes were 79.3–100%. Similarly, the 6 Rep proteins shared high similarity to each other (98.7–99.7%), but low similarity to some of the 24 reference genomes (87.3–100%). Compared with 24 unique reference amino acid sequences, the diversity of amino acid sequences by PCV2 ORF2 in this study was shown in Figure 3. In this study, 15 mutation sites (A47S, M72L, N77D, S86T, P88K, L89I, V130F, T131P, A133S, T151P, L185M, L187I, T190S, G191K and T206K) were identified in PCV2a ORF2 that were absent in PCV2b and PCV2d, ten mutation sites (V57I, A59R, S63K, L89R, T90S, T190A, H200I, A210E, K227R and K234^*^) were identified in PCV2b ORF2 that were absent in PCV2a and PCV2d and 11 substitution sites (Y8F, V30L, F53I, A68N, E104D, F129S, A133T, T134N, S169G/R, V215I and M217L) were identified in PCV2d ORF2 that were absent in PCV2a and PCV2b. Furthermore, there were 69 amino acid mutations finding in the Cap protein of these 35 PCV2 strains, 28 of which occurred in the epitope region reported in previous studies (29).

#### Genetic characteristics of PCV3

In this study, sequence analysis and phylogenetic study of 18 PCV3 strains were performed. The phylogenetic tree was established based on the combined coding sequences (ORF1 + ORF2) of the 18 strains and 25 reference sequences (28). The 43 PCV3 strains contained two major genotypes, PCV3a and PCV3b, among which PCV3a was further divided into PCV3a-1, PCV3a-2 and PCV3a-IM (Figure 2B). In this study, 14 strains of 19 PCV3 were clustered as PCV3a and 4 strains clustered as PCV3b.

The sequence analysis showed that the 18 stains shared 98.7–99.9% identity of their whole genomes, 97.2–100% identity of PRF2 and 97.9–99.9% of ORF1. Then, compared with 25 unique PCV3 whole genomes deposited in the GenBank database, the 18 PCV3 strains shared 99.8–99.9% identity of their whole genomes, 97.1-100% identity of ORF2 and 98.7–99.9% of OFR1. Among the 18 unique PCV3 sequences, 7 unique capsid (Cap) proteins and 8 unique replicase (Rep) proteins were generated based on nucleotide mutations. At the amino acid level, the similarities between the 7 Cap proteins were 97.7–99.5%, while the similarities between them and the 25 PCV3 reference genomes were 95.4–100%. Likewise, the similarity between the 8 Rep proteins was high (98.0–99.7%), while the similarity with the 25 published sequences was low (96.3–100%). The amino acid mutations of the Cap protein of the 18 PCV3 strains sequenced in this study was shown in Supplementary Table S2, which was also compared with 14 unique reference amino acid sequences. Overall, mutations were found in 11 amino acid positions in the Cap protein and in 14 positions in the Rep protein. Among them, there were 3 amino acids (A24V, R27K and E128D) of the Cap protein mutation falling into the antibody recognition domains (23–35aa and 119–131aa within PCV3 Cap protein) as previously reported (30). And there were 7 Cap amino acid mutations (A5P, A24V, T77S, F104Y, E128D, L150I, and T175K) finding in the predicted B-cell epitopes in 18 PCV3 strains sequenced (28).

## Discussion

A total of 257 clinical samples collected from southwest China during 2020–2022 were screened for the prevalence of PCV2 and PCV3. The positive rates of PCV2 in this study was 26.46%, which was higher than previously reported in the Midwest United States and European countries (30, 31). Meanwhile, 43.48% (10/23) and 56.52% (13/23) of the farms were positive for PCV2 and PCV3 respectively, indicating that PCV3 had a higher prevalence found that among the 23 pig farms, and 26.09% (6/23) of the farms were diagnosed as PCV2 and PCV3 coinfection. Among them, 84.62% (11/13) of farms negative for PCV2 were vaccinated against PCV2, while 30% (3/10) of the farms positive for PCV2 were vaccinated. These results showed that routine vaccination helps to prevent the prevalence and the spread of PCV2. Those infected despite vaccination might be caused by immunization failure or by rapid genetic mutation of PCV2. The three farms that failed immunization were inoculated with inactivated PCV2 vaccine, of which 2 farms were inoculated with vaccine (SH) purchased from PULIKE BIO-ENGINEERING Co., Ltd (Henan, China), and the other farm was inoculated with vaccine (DBN-SX07) purchased from HAILINGE BIO-PHARMA CO., Ltd (Chengdu, China). PCV2QL-202012 and PCV2MY-202105 were obtained by sequencing in pig farms inoculated with SH strain. Based on SH strain, amino acid alignment analysis showed that L89R, T90S, and D210E were mutated in the typical motifs, and T200I was mutated in the epitope region. PCV2SN-202101 was detected in pig farms inoculated with DBN, and the A68N and F129S mutations were found at epitope region after comparison. These unique amino acid mutations might be responsible for its immune failure. From 2020 to 2022, PCV2 and PCV3 were detected in 12 cities in Sichuan Province, and the prevalence of both PCV2 and PCV3 is gradually increasing, which indicated that PCV2 and PCV3 are widely distributed in pig farms in the Southwest of China.

In recent years, it has been found that viremia of PCV2 and PCV3 and the manifestation of PCVAD symptoms are often accompanied by mixed infections of the two (32). In previous reports, the coinfection rate of the two in pig farms is generally 27.6-39.39% (33–35). In a research found in the last 2 years, the coinfection rate of PCV2/PCV3 has reached 69.74% in a pig farm in Henan, China, and the main symptoms has been severe watery diarrhea with poor healing (20). Compared with previous data, the overall coinfection rates in Sichuan province (8.95%) in this study were lower than those in central China but higher than those in the Midwest of the USA (19, 30). Thus, necessity is needed to detect the coinfection of PCV2 and PCV3 in China. In the Southwest of China for last 3 years, the coinfection rate has been rising rapidly, and has even doubled from 2020 (5.75%) to 2022 (10.45%). The reason of the lower growth rate for 2021–2022 might be attributed to the lower sample size in 2022 as only the first half of the year was tested. Also, since PCVs prevalence is the highest mainly in winter after weaning (36), the incidence rate in spring 2022 cannot fully reflect the infection rate in that region. In addition, some studies have reported higher rates of infection and more pronounced clinical symptoms of PCV3 when other viruses are present, including PCV2, porcine parvovirus (PPV), and PRRSV (37, 38). In this study, PCV2 and PCV3 were more likely to co-infect with respiratory and enteroviruses, which is generally consistent with the findings in this study (39). However, in another study, PDNS-like clinical cases were replicated in piglets by attacking PCV3 alone (40), which suggests that PCV3, like PCV2, can pose a threat when infected alone.

Recent reports indicate that PCV2 and PCV3 can be detected from various tissues and serum (30). The data in this study showed that PCV2 and PCV3 are more prone to be detected in the spleen and lymph nodes than in other tissues, suggesting that PCV2 mainly colonized in the immune organs and subsequently caused significant depletion of lymphocyte, which was consistent with previous reports (19). The detection rate of circovirus in serum was higher than other tissues but lower than lymph node and spleen, illustrating that the viremia of circovirus was very obvious. Therefore, lymph node, spleen and serum should be considered as important sample types in exploring the prevalence and epidemiology studies of PCVs.

Since PCV2 was detected in 2000 in China, PCV2a has been the most predominant genotype in pig herds until a shift to PCV2b occurred around 2003 (41, 42). Since then, PCV2b has been the most prevalent genotype, and most vaccines has been genotyped PCV2a/2b to date (42). For the past few years, PCV2d has become the main prevalent genotype at home and abroad. In this study, 13 unique PCV2 whole genomes were sequenced. Most of the isolates (53.85%; 7/13) belong to the PCV2d genotype, indicating that it is the main genotype prevalent in southwest China, which is consistent with previous reports (19). Besides, the rates of PCV2a and PCV2b in PCV2-positive samples in this study were 7.69% (1/13) and 38.46% (5/13), respectively.

In phylogenetic genotyping analysis, PCV2 strains showed obvious differences. Among the 13 strains, the genome size of them was 1,767 nucleotide (nt), same as the usual sequence reported earlier. And the length of ORF1 of 13 strains was 945 nt, ORF2 of 4 strains was 702 nt, while ORF2 of the other 9 strains was 705 nt. In previous studies, the PCV2d strain had an extra acid in the C-terminus of it capsid protein compared with PCV2a and PCV2b strains, resulting in a length of 705 nt instead of 702 nt. Interestingly, PCV2MY-202103 in this study belonged to PCV2d, but its capsid protein had no additional amino acids, whilst PCV2QL-202012, PCV2MY-202104, and PCV2MY-202105 (PCV2b) had an extra amino acid, which had also been found in previous studies (19). The amino acid mutation results showed a large number of mutation sites in PCV2. The representative amino acid mutation sites which located in the epitope region were M72L, N77D, S86T, V130F, T131P, A133S, and T206K for PCV2a; H200I and K227R for PCV2b; A68N, S121T and T134N for PCV2d. The immunosuppression of PCV2 might be related to its high amino acid site variability. Nevertheless, the specific mechanisms of PCV2 pathogenicity and immune evasion are not fully understood, so further investigation are highly required.

PCV3 was first detected in 2015 in sows with PDNS and aborted fetuses, and has subsequently been identified in pigs in Asia, Europe, and the Americas (4). The construction of a phylogenetic tree was based on the whole genome sequence of PCV3 and PCV3 can be classified into three genotypes, PCV3a, PCV3b, and PCV3c, of which PCV3a can be subdivided into three genotypes, PCV3a-1, PCV3a-2, and PCV3a-IM (43). In 2019, researches based on 51 PCV3 strains from 21 provinces in China found that the proportion of PCV3a, PCV3b and PCV3c were 29.4, 41.2, and 29.4%, respectively, which showed that PCV3b accounted for the highest proportion and was presumed to be the most prevalent strain in China; PCV3a was mainly prevalent in the Central Plains, PCV3b in the Northeast, and PCV3c was widely distributed in six regions in China, including the North, Northeast, Central Plains, East, Southwest and South (39). In this study, PCV3a accounted for the majority but PCV3b was less prevalent, which was similar to the results reported from Henan Province last year (19), but was inconsistent with the findings from previous years (39), so it can been shown that the prevalent PCV3 strains in China might have shifted in recent years. In this study, homological analysis indicated that the PCV3 strains had a high identity (98.7~99.9%) among genomes, which was agree with many previous studies (44). Therefore, it was difficult to establish a potential genotypic classification of PCV3 which might be related to this.

Based on the phylogenetic analysis and sequence alignment of Rep + Cap gene sequences, all the isolates could be classified into PCV3a and PCV3b. Interestingly, in PCV3b cluster, the 4 strains sequenced in this study with the same capsid protein were detected, and two amino acids (A24V and R27K) were conserved in this group of strains, which were different from other strains. And the two mutations might be related to the mechanism of viral immune escape (16). Moreover, 7 mutations fell within the predicted B-cell epitopes (28), suggesting that this cluster of strains might have different immunogenicity conferred by the Cap protein. The exact mechanism of PCV3 immunogenicity and immune evasion remains unclear, which warrants further study.

In conclusion, PCV2 and PCV3 in Sichuan China from 2020 to 2022 were investigated and the results showed that PCV2 and PCV3 were ubiquitous in the Southwest of China. Furthermore, PCV2d and PCV3a were the most predominant genotype at present. As a common mixed infection, PCV2 and PCV3 are prone to cause serious PCVAD symptoms such as PDNS, PMWS and PRDC, which may bring significant economic losses to the farms. Therefore, it is necessary to take some measures to prevention and control, such as biosafety management and vaccines. Meanwhile, further studies on the invasion mechanism, immune response pathway and interaction between PCV2 and PCV3 should be conducted in the future, which may require more support from epidemiological and pathogenesis studies.

## Data availability statement

The datasets presented in this study can be found in online repositories. The names of the repository/repositories and accession number(s) can be found in the article/Supplementary material.

## Ethics statement

Written informed consent was obtained from animal owners for this study. All experimental procedures were reviewed and approved by the Sichuan Provincial Laboratory Management Committee [License No: SYXK (chuan) 2019–187].

## Author contributions

YY and JW conceived, designed, and performed the experiments. TX, LY, SL, XS, ZX, and LZ contributed to data analysis. YY wrote the manuscript. All authors have read and approved the final version of manuscript.

## Funding

This work was supported by the Porcine Major Science and Technology Project of Sichuan Science and Technology Plan (Project No. 2021ZDZX0010-3), the Sichuan Provincial Department of Science and Technology Rural Area Key R&D Program (Project No. 2020YFN0147), the Key R&D Program of Sichuan Science and Technology Plan (Project No. 2022YFN0007), and the Agricultural Industry Technology System of Sichuan Provincial Department of Agriculture (Project No. CARS-SVDIP).

## Conflict of interest

The authors declare that the research was conducted in the absence of any commercial or financial relationships that could be construed as a potential conflict of interest.

## Publisher's note

All claims expressed in this article are solely those of the authors and do not necessarily represent those of their affiliated organizations, or those of the publisher, the editors and the reviewers. Any product that may be evaluated in this article, or claim that may be made by its manufacturer, is not guaranteed or endorsed by the publisher.

## References

[1] Ouyang T Zhang X Liu X Ren L. Co-infection of swine with porcine circovirus type 2 and other swine viruses. Viruses. (2019) 11:185. 10.3390/v11020185 30795620 PMC6410029

[2] Tischer I Rasch R Tochtermann G. Characterization of papovavirus-and picornavirus-like particles in permanent pig kidney cell lines. Zentralbl Bakteriol Orig A. (1974) 226:153–67. 4151202

[3] Meehan BM McNeilly F Todd D Kennedy S Jewhurst VA Ellis JA. Characterization of novel circovirus DNAs associated with wasting syndromes in pigs. J Gen Virol. (1998) 79:2171–9. 10.1099/0022-1317-79-9-2171 9747726

[4] Phan TG Giannitti F Rossow S Marthaler D Knutson TP Li L. Detection of a novel circovirus PCV3 in pigs with cardiac and multi-systemic inflammation. Virol J. (2016) 13:184. 10.1186/s12985-016-0642-z 27835942 PMC5105309

[5] Xu T Hou CY Zhang YH Li HX Chen XM Pan JJ. Simultaneous detection and genetic characterization of porcine circovirus 2 and 4 in Henan province of China. Gene. (2022) 808:145991. 10.1016/j.gene.2021.145991 34626723

[6] Tischer I Gelderblom H Vettermann W Koch MA A. very small porcine virus with circular single-stranded DNA. Nature. (1982) 295:64–6. 10.1038/295064a0 7057875

[7] Gillespie J Opriessnig T Meng XJ Pelzer K Buechner-Maxwell V. Porcine circovirus type 2 and porcine circovirus-associated disease. J Vet Intern Med. (2009) 23:63. 10.1111/j.1939-1676.2009.0389.x 19780932 PMC7166794

[8] Segales J. Porcine circovirus type 2 (PCV2) infections: clinical signs, pathology and laboratory diagnosis. Virus Res. (2012) 164:10–9. 10.1016/j.virusres.2011.10.007 22056845

[9] Palinski R Piñeyro P Shang P Yuan F Guo R Fang Y. A novel porcine circovirus distantly related to known circoviruses is associated with porcine dermatitis and nephropathy syndrome and reproductive failure. J Virol. (2017) 91:e01879-16. 10.1128/JVI.01879-16 27795441 PMC5165205

[10] Zhang HH Hu WQ Li JY Liu TN Zhou JY Opriessnig T. Novel circovirus species identified in farmed pigs designated as Porcine circovirus 4, Hunan province, China. Transbound Emerg Dis. (2020) 67:1057–61. 10.1111/tbed.13446 31823481

[11] Li W Liu S Wang Y Deng F Yan W Yang K. Transcription analysis of the porcine alveolar macrophage response to porcine circovirus type 2. BMC Genomics. (2013) 14:353. 10.1186/1471-2164-14-353 23711280 PMC3680065

[12] Karuppannan AK Opriessnig T. Porcine circovirus type 2 (PCV2) vaccines in the context of current molecular epidemiology. Viruses. (2017) 9:99. 10.3390/v9050099 28481275 PMC5454412

[13] Franzo G Segalés J. Porcine circovirus 2 (PCV-2) genotype update and proposal of a new genotyping methodology. PLoS ONE. (2018) 13:e0208585. 10.1371/journal.pone.0208585 30521609 PMC6283538

[14] Ouyang T Niu G Liu X Zhang X Zhang Y Ren L. Recent progress on porcine circovirus type 3. Infect Genet Evol. (2019) 73:227–33. 10.1016/j.meegid.2019.05.009 31096019

[15] Tan CY Opaskornkul K Thanawongnuwech R Arshad SS Hassan L Ooi PT. First molecular detection and complete sequence analysis of porcine circovirus type 3 (PCV3) in Peninsular Malaysia. PLoS ONE. (2020) 15:e0235832. 10.1371/journal.pone.0235832 32706778 PMC7380639

[16] Fu X Fang B Ma J Liu Y Bu D Zhou P. Insights into the epidemic characteristics and evolutionary history of the novel porcine circovirus type 3 in southern China. Transbound Emerg Dis. (2018) 65:e296–303. 10.1111/tbed.12752 29178283

[17] Franzo G He W Correa-Fiz F Li G Legnardi M Su S. A shift in porcine circovirus 3 (PCV-3) history paradigm: phylodynamic analyses reveal an ancient origin and prolonged undetected circulation in the worldwide swine population. Adv Sci. (2019) 6:1901004. 10.1002/advs.201901004 31763138 PMC6865002

[18] Chen N Huang Y Ye M Li S Xiao Y Cui B. Co-infection status of classical swine fever virus (CSFV), porcine reproductive and respiratory syndrome virus (PRRSV) and porcine circoviruses (PCV2 and PCV3) in eight regions of China from 2016 to 2018. Infect Genet Evol. (2019) 68:127–35. 10.1016/j.meegid.2018.12.011 30572028

[19] Xu T Zhang YH Tian RB Hou CY Li XS Zheng LL. Prevalence and genetic analysis of porcine circovirus type 2 (PCV2) and type 3 (PCV3) between 2018 and 2020 in central China. Infect Genet Evol. (2021) 94:105016. 10.1016/j.meegid.2021.105016 34325052

[20] Guo Z Ruan H Qiao S Deng R Zhang G. Co-infection status of porcine circoviruses (PCV2 and PCV3) and porcine epidemic diarrhea virus (PEDV) in pigs with watery diarrhea in Henan province, central China. Microb Pathog. (2020) 142:104047. 10.1016/j.micpath.2020.104047 32036077

[21] Lv Q Wang T Deng J Chen Y Yan Q Wang D. Genomic analysis of porcine circovirus type 2 from southern China. Vet Med Sci. (2020) 6:875–89. 10.1002/vms3.288 32510830 PMC7738708

[22] Zhao Y Han HY Fan L Tian RB Cui JT Li JY. Development of a TB green II-based duplex real-time fluorescence quantitative PCR assay for the simultaneous detection of porcine circovirus 2 and 3. Mol Cell Probes. (2019) 45:31–6. 10.1016/j.mcp.2019.04.001 30980890

[23] Ding G Fu Y Li B Chen J Wang J Yin B. Development of a multiplex RT-PCR for the detection of major diarrhoeal viruses in pig herds in China. Transbound Emerg Dis. (2020) 67:678–85. 10.1111/tbed.13385 31597013 PMC7168528

[24] Zheng LL Chai LY Tian RB Zhao Y Chen HY Wang ZY. Simultaneous detection of porcine reproductive and respiratory syndrome virus and porcine circovirus 3 by SYBR Green I-based duplex real-time PCR. Mol Cell Probes. (2020) 49:101474. 10.1016/j.mcp.2019.101474 31655106

[25] Bliss N Nelson SW Nolting JM Bowman AS. Prevalence of influenza a virus in exhibition swine during arrival at agricultural fairs. Zoonoses Public Health. (2016) 63:477–85. 10.1111/zph.12252 26750204 PMC8634047

[26] Moiso N Pieters M Degano F Vissio C Camacho P Estanguet A. Detection of Mycoplasma hyopneumoniae in nasal and laryngeal swab specimens in endemically infected pig herds. Vet Rec. (2020) 186:27. 10.1136/vr.105525 31732508

[27] Gagnon CA Tremblay D Tijssen P Venne MH Houde A Elahi SM. The emergence of porcine circovirus 2b genotype (PCV-2b) in swine in Canada. Can Vet J. (2007) 48:811-9. 17824323 PMC1914312

[28] Li G He W Zhu H Bi Y Wang R Xing G. Origin, genetic diversity, and evolutionary dynamics of novel porcine circovirus 3. Adv Sci. (2018) 5:1800275. 10.1002/advs.201800275 30250786 PMC6145280

[29] Kweon CH Nguyen LT Yoo MS Kang SW. Differential recognition of the ORF2 region in a complete genome sequence of porcine circovirus type 2 (PCV2) isolated from boar bone marrow in Korea. Gene. (2015) 569:308–12. 10.1016/j.gene.2015.04.055 25917618

[30] Wang Y Noll L Lu N Porter E Stoy C Zheng W. Genetic diversity and prevalence of porcine circovirus type 3 (PCV3) and type 2 (PCV2) in the Midwest of the USA during 2016-2018. Transbound Emerg Dis. (2020) 67:1284–94. 10.1111/tbed.13467 31886622

[31] Saporiti V Huerta E Correa-Fiz F Grosse Liesner B Duran O Segalés J. Detection and genotyping of Porcine circovirus 2 (PCV-2) and detection of Porcine circovirus 3 (PCV-3) in sera from fattening pigs of different European countries. Transbound Emerg Dis. (2020) 67:2521–31. 10.1111/tbed.13596 32356364 PMC7754154

[32] Opriessnig T Halbur PG. Concurrent infections are important for expression of porcine circovirus associated disease. Virus Res. (2012) 164:20–32. 10.1016/j.virusres.2011.09.014 21959087 PMC7114432

[33] Li X Qiao M Sun M Tian K. A duplex real-time PCR assay for the simultaneous detection of porcine circovirus 2 and circovirus 3. Virol Sin. (2018) 33:181–6. 10.1007/s12250-018-0025-2 29616412 PMC6178106

[34] Kim HR Park YR Lim DR Park MJ Park JY Kim SH. Multiplex real-time polymerase chain reaction for the differential detection of porcine circovirus 2 and 3. J Virol Methods. (2017) 250:11–6. 10.1016/j.jviromet.2017.09.021 28941615

[35] Zheng S Wu X Zhang L Xin C Liu Y Shi J. The occurrence of porcine circovirus 3 without clinical infection signs in Shandong Province. Transbound Emerg Dis. (2017) 64:1337–41. 10.1111/tbed.12667 28653486 PMC7169790

[36] Vangroenweghe F Thas O. Seasonal variation in prevalence of mycoplasma hyopneumoniae and other respiratory pathogens in peri-weaned, post-weaned, and fattening pigs with clinical signs of respiratory diseases in Belgian and Dutch pig herds, using a tracheobronchial swab sampling technique, and their associations with local weather conditions. Pathogens. (2021) 10:1202. 10.3390/pathogens10091202 34578234 PMC8471121

[37] Sukmak M Thanantong N Poolperm P Boonsoongnern A Ratanavanichrojn N Jirawattanapong P. The retrospective identification and molecular epidemiology of porcine circovirus type 3 (PCV3) in swine in Thailand from 2006 to 2017. Transbound Emerg Dis. (2019) 66:611–6. 10.1111/tbed.13057 30387296

[38] Ha Z Xie CZ Li JF Wen SB Zhang KL Nan FL. Molecular detection and genomic characterization of porcine circovirus 3 in pigs from Northeast China. BMC Vet Res. (2018) 14:321. 10.1186/s12917-018-1634-6 30367641 PMC6203981

[39] Qi S Su M Guo D Li C Wei S Feng L. Molecular detection and phylogenetic analysis of porcine circovirus type 3 in 21 Provinces of China during 2015-2017. Transbound Emerg Dis. (2019) 66:1004–15. 10.1111/tbed.13125 30637986

[40] Jiang H Wang D Wang J Zhu S She R Ren X. Induction of porcine dermatitis and nephropathy syndrome in piglets by infection with porcine circovirus type 3. J Virol. (2019) 93:e02045-18. 10.1128/JVI.02045-18 30487279 PMC6363995

[41] Lang H Zhang G Wu F Zhang C. Detection of serum antibody against postweaning multisystemic wasting syndrome in pigs. Chin J Vet Technol. (2000) 3:3–5. 10.16656/j.issn.1673-4696.2000.03.001

[42] Xu PL Zhao Y Zheng HH Tian RB Han HY Chen HY. Analysis of genetic variation of porcine circovirus type 2 within pig populations in central China. Arch Virol. (2019) 164:1445–51. 10.1007/s00705-019-04205-0 30888560

[43] Franzo G Delwart E Fux R Hause B Su S Zhou J. Genotyping porcine circovirus 3 (PCV-3) nowadays: does it make sense? Viruses. (2020) 12:265. 10.3390/v12030265 32121102 PMC7150946

[44] Klaumann F Correa-Fiz F Franzo G Sibila M Núñez JI Segalés J. Current knowledge on porcine circovirus 3 (PCV-3): a novel virus with a yet unknown impact on the swine industry. Front Vet Sci. (2018) 5:315. 10.3389/fvets.2018.00315 30631769 PMC6315159

