# Supplementary material for: Prevalence and phylogenetic analysis of porcine circovirus type 2 (PCV2) and type 3 (PCV3) in the Southwest of China during 2020–2022
Source: Front Vet Sci. 2022 Nov 24;9:1042792. doi: 10.3389/fvets.2022.1042792 (PMC9731358; doi:10.3389/fvets.2022.1042792)
Supplement: Supplementary file 1 [file Table_1.DOCX]

**Table S1** The information of PCV2 and PCV3 strains for sequence alignment and phylogenetic analysis

| **PCV2 strain** | **Accession number** | **Origin** | **PCV2 reference strain** | **Accession number** | **Origin** | **PCV3 strain** | **Accession number** | **Origin** | **PCV3 reference strain** | **Accession number** | **Origin** |
| --- | --- | --- | --- | --- | --- | --- | --- | --- | --- | --- | --- |
| **PCV2MY-202011** | OP055737 | MY, Sichuan, China, 2020 | HN-BF-2016 | MK604479 | China,2016 | **PCV3NC-202009** | OP066319 | NC, Sichuan, China, 2020 | PCV3-US/MO2015 | KX778720 | USA,2015 |
| **PCV2QL-202011** | OP055738 | QL, Sichuan, China, 2020 | HN-QY-2016 | MK604495 | China,2016 | **PCV3DY-202010** | OP066315 | DY, Sichuan, China, 2020 | PCV3/CN/Fujian-5/2016 | KY075986 | China,2016 |
| **PCV2MY-202012** | OP055739 | MY, Sichuan, China, 2020 | pmws PCV | AF027217 | Canada,1997 | **PCV3QL-202011** | OP066314 | QL, Sichuan, China, 2020 | PCV3/CN/Fujian-12/2016 | KY075987 | China,2016 |
| **PCV2QL-202012** | OP055741 | QL, Sichuan, China, 2020 | Porcine circovirus Type II | AF055392 | Canada,1998 | **PCV3LS-202012** | ON586581 | LS, Sichuan, China, 2020 | PCV3-CN-Henan-13-2016 | KY075988 | China,2016 |
| **PCV2ZG-202012** | OP055746 | ZG, Sichuan, China, 2020 | GX0601 | EF524532 | China,2007 | **PCV3SN-202101** | ON989005 | SN, Sichuan, China, 2021 | PCV3/CN/Jiangxi-62/2016 | KY075989 | China,2016 |
| **PCV2SN-202101** | OP055740 | SN, Sichuan, China, 2021 | LG | HM038034 | China,2008 | **PCV3QL-202101** | OP066316 | QL, Sichuan, China, 2021 | PCV3/CN/Chongqing-147/2016 | KY075990 | China,2016 |
| **PCV2MY-202103** | OP055742 | MY, Sichuan, China, 2021 | 10JS-2 | JQ806749 | China,2010 | **PCV3ZG-202103** | OP066317 | ZG, Sichuan, China, 2021 | PCV3/CN/Chongqing-148/2016 | KY075991 | China,2016 |
| **PCV2MY-202104** | OP055743 | MY, Sichuan, China, 2021 | unknow | AY177626 | China,2002 | **PCV3SN-202103** | OP066323 | SN, Sichuan, China, 2021 | CN/Hubei-610/2016 | KY354038 | China,2016 |
| **PCV3MY-202105** | OP055747 | MY, Sichuan, China, 2021 | P2425NT | JX099786 | Viet Nam,2008 | **PCV3LS-202106** | OP066318 | LS, Sichuan, China, 2021 | CN/Hubei-618/2016 | KY354039 | China,2016 |
| **PCV2NC-202106** | OP055744 | NC, Sichuan, China, 2021 | Porcine circovirus Type II | AF055394 | France,1998 | **PCV3MY-202108** | OP066321 | MY, Sichuan, China, 2021 | CHN_Shanghai_0708_2016 | KY865243 | China,2016 |
| **PCV2DY-202107** | OP055748 | DY, Sichuan, China, 2021 | DBN-SX07-2 | HM641752 | China,2007 | **PCV3MY-202109** | OP066322 | MY, Sichuan, China, 2021 | PCV3/KU-1601 | KY996337 | South Korea,2016 |
| **PCV2PZ-202110** | OP055745 | PZ, Sichuan, China, 2021 | DK1980PMWSfree | EU148503 | Denmark,1980 | **PCV3QL-202111** | OP066328 | QL, Sichuan, China, 2021 | PCV3/KU-1602 | KY996338 | South Korea,2016 |
| **PCV2MY-202205** | OP189294 | MY, Sichuan, China, 2022 | TJ | AY181946 | China,2002 | **PCV3SN-202112** | OP066324 | SN, Sichuan, China, 2021 | PCV3/KU-1604 | KY996340 | South Korea,2016 |
|  |  |  | SH | AY686763 | China,2004 | **PCV3MY-202112** | OP066325 | MY, Sichuan, China, 2021 | PCV3/KU-1605 | KY996341 | South Korea,2016 |
|  |  |  | 22664-35 | JX535297 | USA,2012 | **PCV3ZG-202201** | OP066326 | ZG, Sichuan, China, 2022 | PCV3/KU-1606 | KY996342 | South Korea,2016 |
|  |  |  | KU-1607 | KX828234 | South Korea,2016 | **PCV3MY-202201** | OP066327 | MY, Sichuan, China, 2022 | PCV3-BR/RS/6 | MF079253 | Brazil,2016 |
|  |  |  | MEX/41238/2014 | KT795287 | Mexico,2014 | **PCV3QL-202202** | OP066329 | QL, Sichuan, China, 2022 | PCV3-BR/RS/8 | MF079254 | Brazil,2016 |
|  |  |  | USA/45358/2015 | KT795290 | USA,2015 |  |  |  | PCV3-China/GX2016-3 | MF155643 | China,2016 |
|  |  |  | YN-8 | HM776452 | China,2009 |  |  |  | PCV3-IT/CO2017 | MF162298 | Italy,2017 |
|  |  |  | LN6/1999 | MF278777 | China,1999 |  |  |  | PCV3-IT/MN2017 | MF162299 | Italy,2017 |
|  |  |  | NAVET | JX506730 | Viet Nam,2004 |  |  |  | PCV3/CN/GDBL1/2017 | MF405272 | China,2017 |
|  |  |  | DK1987PMWSfree | EU148504 | Denmark, 1987 |  |  |  | PCV3/CN/Guangdong-HZ4/2015 | MF589103 | China,2015 |
|  |  |  |  |  |  |  |  |  | PCV3/CN/Guangdong-MX3/2015 | MF589104 | China,2015 |
|  |  |  |  |  |  |  |  |  | PCK3-1701 | MF611876 | South Korea,2016 |
|  |  |  |  |  |  |  |  |  | PCK3-1702 | MF611877 | South Korea,2016 |

Note: UN, Unknow. The 13 PCV2 strains and 18 PCV4 strains sequenced in this study are in bold.

Abbreviations: MY, Mianyang; QL,Qionglai; SN, Suining; NC,Nanchong; DY, Deyang; PZ,Pengzhou; LS,Leshan; ZG,Zigong
