# Supplementary material for: Prevalence and phylogenetic analysis of porcine circovirus type 2 (PCV2) and type 3 (PCV3) in the Southwest of China during 2020–2022
Source: Front Vet Sci. 2022 Nov 24;9:1042792. doi: 10.3389/fvets.2022.1042792 (PMC9731358; doi:10.3389/fvets.2022.1042792)
Supplement: Supplementary file 2 [file Table_2.DOCX]

**Table S2** Amino acid mutations of the Cap protein of the 18 PCV3 strains sequenced in this study and 14 unique PCV3 reference strains

| Strain | Positions of Cap amino acid point mutations (position of alignment) | | | | | | | | | | |
| --- | --- | --- | --- | --- | --- | --- | --- | --- | --- | --- | --- |
|  | 5 | 20 | 24 | 27 | 77 | 98 | 104 | 128 | 132 | 150 | 175 |
| PCV3-KU-1604-2016 | A | R | A | R | T | Q | F | E | R | L | T |
| PCV3-CN-GDBL1-2017 | . | . | V | K | S | . | . | . | . | I | . |
| CN-Hubei-618-2016 | . | . | . | . | S | . | . | . | . | I | . |
| PCV3KU-16012016 | . | . | V | . | S | . | . | . | . | I | . |
| PCV3-US-MO-2015 | . | . | . | . | S | . | . | . | . | I | . |
| PCV3-China-GX2016-3 | . | . | . | . | S | . | . | . | . | I | . |
| PCV3-CN-Guangdong-MX3-2015 | . | . | V | K | S | . | . | . | . | I | . |
| PCV3-BR-RS-6-2016 | . | . | . | . | . | . | . | . | . | . | . |
| PCV3-CN-Guangdong-HZ4-2015 | . | . | V | K | S | K | . | . | . | I | . |
| PCV3-KU-1606-2016 | . | K | V | K | S | . | . | . | . | I | . |
| PCV3-KU-1605-2016 | . | . | V | K | S | . | . | . | . | I | . |
| PCK3-1702-2016 | . | . | . | . | . | . | . | . | G | . | . |
| PCV3-IT-CO-2017 | . | . | V | K | S | . | . | . | . | I | . |
| CHN Shanghai 0708-2016 | . | . | V | K | N | . | . | . | . | I | . |
| **PCV3DY-202010** | . | . | . | . | . | . | . | D | . | . | . |
| **PCV3LS-202012** | . | . | V | K | S | . | . | . | . | I | . |
| **PCV3LS-202106** | . | . | . | . | . | . | . | . | . | . | . |
| **PCV3MY-202108** | . | . | . | . | . | . | . | . | . | . | . |
| **PCV3MY-202109** | . | . | . | . | . | . | . | . | . | . | . |
| **PCV3MY-202112** | . | . | . | . | S | . | . | . | . | . | . |
| **PCV3MY-202201** | . | . | V | K | S | . | . | . | . | . | . |
| **PCV3NC-202009** | . | . | . | . | . | . | . | . | . | . | . |
| **PCV3PZ-202112** | . | . | . | . | . | . | . | . | . | . | . |
| **PCV3QL-202011** | . | . | V | K | S | . | . | . | . | I | . |
| **PCV3QL-202101** | . | . | . | . | . | . | . | . | . | . | . |
| **PCV3QL-202111** | . | . | . | . | . | . | . | . | . | . | . |
| **PCV3QL-202202** | . | . | . | . | . | . | . | . | . | . | . |
| **PCV3SN-202101** | . | . | . | . | . | . | . | . | . | . | . |
| **PCV3SN-202103** | . | . | . | . | S | . | Y | . | . | I | . |
| **PCV3SN-202112** | P | . | . | . | S | . | . | . | . | . | K |
| **PCV3ZG-202103** | . | . | . | . | . | . | . | . | . | . | . |
| **PCV3ZG-202201** | . | . | V | K | S | . | . | . | . | I | . |

Note: The subtypes were proposed by Li et al. (2018). The 18 PCV3 strains sequenced in this study are in bold. The blue areas show the antibody recognition domains (Wang el al, 2019). Sequences obtained from this study are in bold. The Chinese strains are marked with gray shading.
